# Bridging the heart–mind divide: Psychiatric implementation of the 2025 ESC mental–CVD consensus

**DOI:** 10.1192/j.eurpsy.2026.10155

**Published:** 2026-01-23

**Authors:** Tommaso Barlattani, Damiano Venturiello, Davide Grassi, Silvio Romano, Francesca Pacitti

**Affiliations:** 1 Department of Biotechnological and Applied Clinical Sciences (DISCAB), University of L’Aquila, L’Aquila, Italy; 2 Division of Cardiology, Department of Clinical and Molecular Medicine, Sapienza University of Rome, Sant’Andrea Hospital, Rome, Italy; 3 Department of Internal Medicine and Public Health, University of L’Aquila, L’Aquila, Italy; 4 Cardiology, Department of Life, Health and Environmental Sciences, University of L’Aquila, L’Aquila, Italy

**Keywords:** cardiac disease-induced post-traumatic stress disorder, cardiac rehabilitation, cardiovascular disease, integrated care, psychopharmacology, severe mental illness, social prescribing

## Abstract

The 2025 ESC (European Society of Cardiology) Clinical Consensus Statement on mental health and cardiovascular disease is a milestone for psychiatry as much as for cardiology. It recognizes mental disorders as major determinants of cardiovascular (CV) risk and explicitly calls for collaboration with the European Psychiatric Association (EPA). In parallel, the EPA Presidential Action Plan and its “Whole Person Health” task force promote lifestyle‑based, multimorbidity-focused care. From a psychiatric perspective, the challenge is now to translate these frameworks into everyday practice. In this Viewpoint, we propose three priorities. First, severe mental illness (SMI) and cardiac disease-induced post-traumatic stress disorder (CDI-PTSD) should be treated as high‑risk conditions that trigger proactive CV assessment and structured follow‑up. Second, mental‑health services should adopt a simple “safety bundle” for psychotropic medications in people with, or at high risk of, CV disease. Third, psychiatrists should use cardiac rehabilitation, structured physical activity and social prescribing as psychiatric interventions.

## A psychiatric reading of ESC consensus and EPA “Whole Person Health”

The 2025 ESC consensus on mental health and cardiovascular diseases highlights that mental disorders play a critical role in CV outcomes. [1]. The paper emphasizes the strong evidence that SMI and common mental disorders are associated with an increased risk of CV disease and worsen prognosis once CV disease is present [1, 2].

At the same time, the EPA Presidential Action Plan has set out a roadmap for better and personalized mental-health care in Europe, including a dedicated task force on lifestyle, multimorbidity and “Whole Person Health” [3, 4]. This task force aims to integrate physical and mental health, strengthen collaboration with other medical specialties (including cardiology), promote lifestyle-based interventions, and reduce premature mortality among people with SMI.

Our Viewpoint can be seen as a cardiovascular test case of this agenda. We ask: what would it mean, in practical terms, for psychiatrists to implement the ESC Consensus on mental health and CV disease within a Whole Person Health framework? We propose three priorities: treating SMI and CDI‑PTSD as high-risk conditions that trigger CV assessment; adopting a pragmatic psychotropic safety bundle; and using cardiac rehabilitation, structured physical activity, and social prescribing as psychiatric treatment platforms.

## Severe mental illness and CDI‑PTSD as cardiovascular high-risk conditions

People with SMI, including schizophrenia spectrum disorders, bipolar disorder, and recurrent major depression with marked functional impairment, die 10–15 years earlier than the general population, largely because of preventable somatic conditions, especially CV disease [1, 2, 5]. This mortality gap is driven by modifiable factors: tobacco use, physical inactivity, poor diet, psychotropic side‑effects, diagnostic overshadowing, and barriers to guideline-concordant somatic care.

The ESC Consensus calls for systematic mental-health screening in cardiology settings [1]. From the psychiatric point of view, an equally strong commitment is needed in the opposite direction: SMI should itself trigger basic CV risk assessment and, when indicated, referral to primary care or cardiology.

Psychiatrists and community mental health teams play an important role in monitoring patients with severe mental illness (SMI) by checking vital health metrics like blood pressure, smoking habits, weight, waist circumference, and essential lab markers such as lipid levels and glucose/HbA1c. They can gain important information about a patient’s health by incorporating simple cardiovascular risk assessment tools into their routine care. However, it’s important to remember that these tools might not fully capture the risk for patients with SMI, so clinicians should approach the results with that understanding.

Additionally, it is important to establish clear referral pathways for somatic care when any abnormalities are detected.

Key “sentinel” moments for such screening include first-episode psychosis, initiation or escalation of antipsychotics with high metabolic risk, mood stabilizer titration, and discharge after psychiatric hospitalization. At each of these points, a brief CV assessment can be embedded into standard psychiatric workflows, with agreed feedback loops from primary care and cardiology.

The ESC document also devotes substantial attention to post-traumatic stress disorder (PTSD) as both a risk factor and a consequence of CV disease [1]. A growing literature describes CDI‑PTSD, in which PTSD symptoms follow a life-threatening cardiac event such as myocardial infarction, cardiac arrest, spontaneous coronary artery dissection, heart transplantation, or device shocks [6].

Clinically, CDI‑PTSD is characterized by re-experiencing of the event and its bodily sensations (palpitations, chest pain, dyspnea), avoidance of exertion and medical settings, hyperarousal with cardiac-focused anxiety, and behavioral patterns that undermine adherence to medication, lifestyle change, and rehabilitation [6, 7]. Physiologically, PTSD contributes to CV risk through autonomic imbalance, systemic inflammation, and unhealthy coping behaviors [7]. Behaviorally, CDI‑PTSD is marked by persistent avoidance of physical activity, which the ESC Consensus highlights as a major driver of adverse prognosis [1].

We suggest that CDI‑PTSD should be explicitly recognized in psychiatric training and local protocols. One of the approach’s practical implications is the implementation of systematic PTSD screenings for patients who have had acute cardiovascular events, whether in emergency rooms, outpatient settings, or through liaison services. It recommends establishing cooperative channels between cardiology and psychology (psycho-cardio teams) to deliver quick trauma-focused therapies. Additionally, it highlights the importance of integrating graded exposure to physical activity into cardiac rehabilitation programs. This aims to help patients manage their fear of exertion, addressing this psychological aspect as an important part of their recovery.

## A psychotropic safety bundle for people with cardiovascular disease

Psychotropic medications remain essential for treating many mental disorders, but their cardiometabolic and electrophysiological side‑effect profiles vary widely [1, 5]. Antipsychotics such as clozapine and olanzapine are strongly associated with weight gain, dyslipidemia, and dysglycaemia; several antidepressants and antipsychotics can prolong the QT interval, especially in combination with antiarrhythmics or other QT-prolonging drugs [1, 5].

Evidence-based recommendations exist, yet implementation is patchy. We propose that psychiatric services adopt a simple safety bundle whenever patients have, or are at high risk of, CV disease:

Baseline and follow-up cardiometabolic assessment

- Measure weight, body‑mass index, waist circumference, blood pressure, fasting glucose or HbA1c, and lipid profile at baseline when initiating or switching higher-risk psychotropics.
- Repeat at 3 and 6 months, then at least annually, with more frequent monitoring in high-risk patients.

ECG and interaction checks for QT-prolonging or arrhythmogenic drugs

- Obtain baseline and early follow-up ECGs for psychotropics known to prolong QTc or interact with CV medications. Depending on the patient’s cardiovascular risk of developing torsade de pointes and QT prolongation, the rate of QTc monitoring should be modified. While patients at lower risk may be monitored at longer intervals, those at higher risk need more frequent ECG surveillance. [8].
- Routinely check for drug–drug interactions and consult cardiology when combining multiple QT-prolonging drugs or antiarrhythmics.

Shared decision-making and early switching

- Discuss benefits and risks, including CV risk, when choosing antipsychotics or antidepressants.
- Be prepared to switch to agents with lower metabolic or arrhythmic risk when clinically feasible, rather than accepting substantial weight gain or QTc changes as inevitable.

This bundle can be implemented by psychiatrists and mental-health nurses, with cardiology input as needed, and fits squarely within the Whole Person Health task force’s call to embed cardiometabolic monitoring and lifestyle-related practices into psychiatric care [3, 4].

## Cardiac rehabilitation, physical activity, and social prescribing as psychiatric interventions

Cardiac rehabilitation and long‑term CV follow-up are usually seen as cardiology domains, but they are also under‑used psychiatric treatment settings. The ESC Consensus calls for systematic mental-health assessment in cardiac rehabilitation [1]. EPA guidance on physical activity for SMI goes further, showing that structured physical activity is an effective treatment for depressive symptoms, negative symptoms, and functional impairment in schizophrenia, major depression, and bipolar disorder [9]. EPA recommends at least 150 minutes of moderate‑intensity or 75 minutes of vigorous‑intensity activity per week, plus resistance training on 2 days per week, adapted to individual capacity [9].

An EPA meta‑review confirms that multi-component lifestyle interventions combining exercise, diet, and smoking cessation can improve cardiometabolic outcomes in adults with SMI [7]. These findings underpin the EPA Whole Person Health task force, which explicitly prioritizes physical activity, nutrition, sleep, and social connectedness as levers to reduce premature mortality [3, 4]. Within this framework, psychiatrists should regard physical activity not merely as a “healthy habit” but as a core psychiatric intervention with robust evidence.

Working with cardiology and rehabilitation teams, psychiatrists can ensure that people with SMI are actively referred to, and supported in, accessing cardiac rehabilitation when indicated; contribute psychological input focusing on motivation, adherence and fear of exertion; and use rehabilitation as a supervised environment to deliver EPA-concordant physical‑activity prescriptions, including graded exposure for patients with CDI‑PTSD.

The ESC consensus also highlights social prescribing – referrals to community-based activities such as walking groups, arts and music programs, gardening, green exercise, and peer‑support groups [1]. Systematic reviews show that social prescribing can improve depressive and anxiety symptoms, well-being, and quality of life, especially when link workers coordinate care [10], while nature-based prescriptions may additionally reduce blood pressure and improve cardiometabolic indicators [10].

Psychiatry has a long tradition of community-based psychosocial interventions; social prescribing offers a practical framework to reconnect this tradition with CV prevention. In collaboration with primary care and social‑care services, psychiatrists can identify patients with SMI, CDI-PTSD, or cardiac anxiety who may benefit from community activities, work with link workers to match people to programs, and monitor mental‑health outcomes as part of routine follow-up. Digital platforms can support social prescribing and activity monitoring, but should always be complemented by non-digital options and active support to avoid excluding older or disadvantaged groups [10].

## Conclusion

Seen from a psychiatric perspective, the 2025 ESC Clinical Consensus Statement is more than a cardiology document; together with the EPA Presidential Action Plan and the Whole Person Health task force, it is an invitation to reshape how mental-health services understand and manage CV risk [1, 3, 4]. By treating SMI and CDI‑PTSD as high-risk conditions that demand structured CV assessment, adopting pragmatic safety bundles for psychotropic medications, and embracing cardiac rehabilitation, structured physical activity, and social prescribing as psychiatric interventions, psychiatry can begin to operationalize Whole Person Health in the cardiovascular domain and move from describing the mortality gap to systematically closing it.

## Data Availability

Not applicable. No new data were created or analysed.

## References

[1] Bueno H, Deaton C, Farrero M, Forsyth F, Braunschweig F, Buccheri S, et al. ESC clinical consensus statement on mental health and cardiovascular disease: developed under the auspices of the ESC clinical practice guidelines committee. Eur Heart J. 2025 Nov 3;46(41):4156–225. doi:10.1093/eurheartj/ehaf191. 40878270

[2] Shen Q, Mikkelsen DH, Luitva LB, Song H, Kasela S, Aspelund T, et al. Psychiatric disorders and subsequent risk of cardiovascular disease: a longitudinal matched cohort study across three countries. EClinicalMedicine. 2023;61:102063. doi:10.1016/j.eclinm.2023.102063. 37425374 PMC10329128

[3] Fiorillo A, Firth J, Misiak B, Rojnic Kuzman M, Samochowiec J, Sampogna G, et al. From lifestyle psychiatry to whole person health: evidence, dissemination and scalable approaches. Eur Psychiatry 2025 Nov 18:1–10. doi:10.1192/j.eurpsy.2025.10133. Epub ahead of print. PMC12925668 41249118

[4] Fiorillo A. A roadmap for better and personalized mental health care in Europe – the action plan of the European psychiatric association. Eur Psychiatry. 2025;68(1):1–3, e60. doi:10.1192/j.eurpsy.2025.2456. PMC12188326 40405767

[5] Correll CU, Solmi M, Veronese N, Bortolato B, Rosson S, Santonastaso P, et al. Prevalence, incidence and mortality from cardiovascular disease in patients with pooled and specific severe mental illness: a large‑scale meta‑ analysis of 3,211,768 patients and 113,383,368 controls. World Psychiatry. 2017;16(2):163–80. doi:10.1002/wps.20420; Erratum in: World Psychiatry. 2018 Feb;17(1):120. doi:10.1002/wps.20503. 28498599 PMC5428179

[6] Edmondson D, Cohen BE. Posttraumatic stress disorder and cardiovascular disease. Prog Cardiovasc Dis. 2013;55(6):548–56. doi:10.1016/j.pcad.2013.03.004. 23621964 PMC3639489

[7] Maurus I, Wagner S, Spaeth J, Vogel J, Muenz A, Seitz S, et al. EPA guidance on lifestyle interventions for adults with severe mental illness: a meta-review of the evidence. Eur Psychiatry. 2024;67(1):e80. doi:10.1192/j.eurpsy.2024.1766. 39655999 PMC11733621

[8] Zolezzi M, Cheung L. A literature-based algorithm for the assessment, management, and monitoring of drug-induced QTc prolongation in the psychiatric population. Neuropsychiatr Dis Treat. 2018;15:105–14. doi:10.2147/NDT.S186474. 30636876 PMC6309020

[9] Stubbs B, Vancampfort D, Hallgren M, Firth J, Veronese N, Solmi M, et al. EPA guidance on physical activity as a treatment for severe mental illness: a meta-review of the evidence and position statement from the European psychiatric association (EPA), supported by the International Organization of Physical Therapists in mental health (IOPTMH). Eur Psychiatry. 2018;54:124–44. doi:10.1016/j.eurpsy.2018.07.004. 30257806

[10] Nguyen PY, AstellBurt T, RahimiArdabili H, Feng X. Effect of nature prescriptions on cardiometabolic and mental health, and physical activity: a systematic review. Lancet Planet Health. 2023;7(4):e313–28. doi:10.1016/S2542-5196(23)00025-6. 37019572

